# Supplementary material for: Sex as a Predictor of Response to Immunotherapy in Advanced Cutaneous Squamous Cell Carcinoma
Source: Cancers (Basel). 2023 Oct 17;15(20):5026. doi: 10.3390/cancers15205026 (PMC10605413; doi:10.3390/cancers15205026)
Supplement: Supplementary file 1 [file cancers-15-05026-s001.zip › Table S1 - Univariate and multivariate OS analysis.pdf]

Supplementary Table S1: Univariate and multivariate OS analysis

| | Univariate | | | Multivariate | | |
|---|---|---|---|---|---|---|
| | **HR** | **95% CI** | **P** | **HR** | **95% CI** | **P** |
| **Age** | | | | | | |
| < 69 | 1 | | 0.007 | 1 | | 0.14 |
| 69 - <79 | 4.0 | 0.8 – 21.1 | | 3.3 | 0.6 – 18.1 | |
| >= 79 | 6.9 | 1.6 – 30.7 | | 5.0 | 0.9 – 25.3 | |
| **Sex** | | | | | | |
| Male | 1 | | | 1 | | |
| Female | 3.0 | 1.3 – 7.1 | 0.006 | 2.5 | 1.1– 6.1 | 0.03 |
| **CCI** | | | | | | |
| <5 | 1 | | 0.02 | 1 | | 0.41 |
| 5 or more | 3.1 | 1.1 – 8.4 | | 5.1 | 1.1 – 23.7 | |
| **Site of Primary SCC** | | | | | | |
| Head and neck | 1 | | 0.91 | | | |
| Other | 1.0 | 0.4 – 2.5 | | | | |
| **Disease extent** | | | 0.98 | | | |
| Locoregional | 1 | | | | | |
| metastatic | 1.0 | 0.4 – 2.4 | | | | |
| **Visceral Mets** | | | 0.57 | | | |
| No | 1 | | | | | |
| Yes | 0.7 | 0.2 – 2.2 | | | | |
| **Significant Immunosuppression** | | | 0.30 | | | |
| No | 1 | | | | | |
| Yes | 1.7 | 0.6 – 5.2 | | | | |
| **Previous Radiotherapy** | | | 0.85 | | | |
| No | 1 | | | | | |
| Yes | 0.9 | 0.4 – 2.2 | | | | |
| **Previous Surgery for advanced disease** | | | 0.37 | | | |
| No | 1 | | | | | |
| Yes | 0.7 | 0.3 – 1.6 | | | | |
| **Elevated LDH** | | | 0.73 | | | |
| no | 1 | | | | | |
| yes | 1.1 | 0.4 – 3.2 | | | | |
| **Immunotherapy agent** | | | 0.10 | | | |
| Cemiplimab | 1 | | | | | |
| Other agents | 2.1 | 0.8 – 5.4 | | | | |
| **Antibiotics** | | | | | | |
| No | 1 | | 0.39 | | | |
| Yes | 0.53 | 0.1 – 2.3 | | | | |
