# Supplementary material for: Sex as a Predictor of Response to Immunotherapy in Advanced Cutaneous Squamous Cell Carcinoma
Source: Cancers (Basel). 2023 Oct 17;15(20):5026. doi: 10.3390/cancers15205026 (PMC10605413; doi:10.3390/cancers15205026)
Supplement: Supplementary file 1 [file cancers-15-05026-s001.zip › Table S2 - Association between sex and clinical factors.pdf]

Supplementary Table S2: Association between sex and clinical factors

	Total	Male (34)	Female (17)	P (Chi Sq)
**Response**				
Responder	34	29	5	<0.0001
Non Responder	17	5	12	
**Age:**				
< 69	15	12	3	0.37
69 - <79	12	8	4	
>= 79	24	14	10	
**CCI**				
CCI <5	21	15	6	0.54
CCI 5 or more	30	19	11	
**Site of Primary SCC**				
Head and neck	29	22	1	0.10
Other	22	12	10	
**Disease extent**				
Locally advanced	23	16	7	0.69
Metastatic	28	18	10	
Presence of visceral metastases	18	14	4	0.04
**Significant Immunosuppression**				
No	45	31	14	0.35
Yes	6	3	3	
**Antibiotics Prior to Starting ICI**				
No	43	28	15	0.58
Yes	8	6	2	
**Previous Radiotherapy**				
No	22	12	10	0.10

Yes	29	22	7	
**Previous Surgery for advanced disease**				
No	14	6	8	0.02
Yes	37	28	9	
**Elevated LDH**	9	7	2	0.43
**Immunotherapy agent**				
Cemiplimab	21	14	7	0.99
Other	30	20	1	
